# Reversal of High dietary fructose-induced PPARα suppression by oral administration of lipoxygenase/cyclooxygenase inhibitors

**DOI:** 10.1186/1743-7075-2-18

**Published:** 2005-08-09

**Authors:** Glen L Kelley, Salman Azhar

**Affiliations:** 1 Insmed Incorporated, Richmond, VA, USA; 2 Geriatric Research, Education and Clinical Center, VA Palo Alto Health Care System & Stanford University School of Medicine, Palo Alto, CA, USA

## Abstract

High fructose feeding causes diet-induced alterations of lipid metabolism and decreased insulin sensitivity, hallmark of which is a rapid and profound hypertriglyceridemia. One of the mechanisms that contribute to serum hypertriglyceridemia in this model is suppression of hepatic PPARα. HMG-CoA inhibitors, which reduce serum triglycerides in these animals, also elevate/restore hepatic PPARα. Previously we demonstrated that two known lipoxygenase/cyclooxygenase inhibitors reversed diet-induced hypertriglyceridemia in this model and that reversal of certain inflammatory markers in the liver correlated with the metabolic benefit. In this paper we extended these studies by examining the impact of these compounds on expression of PPARα, both at the level of transcription and expression. Our data show that diet-induced suppression of hepaic PPARα is reversed upon treatment with lipoxygenase/cyclooxygenase compounds. We then tested one of these compounds, BW-755c, over a range of doses from 10 mg/kg to 100 mg/kg to establish a dose-response relationship with the reduction of serum hypertriglyceridemia in this model. These experiments support the concept of using anti-inflammatory medications as one method to correct metabolic dysfunction.

## Background

Recent epidemiological studies have shown that almost a quarter of adults in the United States have metabolic syndrome or syndrome X and prevalence of this syndrome is increasing world-wide owing to lifestyle changes leading to obesity [1-3]. A cluster of abnormalities define metabolic syndrome including insulin resistance, hypertriglyceridemia, low high-density lipoprotein (HDL) cholesterol, obesity and hypertension [4-6]; individuals with syndrome have an increased risk of developing cardiovascular disease [5,6]. Insulin resistance is now considered a central factor among these various abnormalities associated with the metabolic syndrome. With this in mind, much effort is being invested in improving the insulin resistance through lifestyle modification (e.g., weight reduction, dietary interventions, and increased physical activity) and development of new therapeutic agents that sensitize insulin action, ameliorate hypertriglyceridemia, raise HDL levels and improve hypertension [2,7-12].

In an animal model, high fructose fed (HFF) diets induce metabolic dysfunction typically resulting in a rapid elevation of serum triglycerides with a corresponding increase in blood pressure within two weeks of diet intitiation. Animals maintained on this diet for longer periods of time develop elevated free fatty acids and hyperinsulinemia at the expense of glycemic control. If HFF animals are subjected to an exercise regimen, the diet-induced effects can be ameliorated [13]. Thus this animal model exhibits an early stage of the Metabolic Syndrome in which a combination of physical inactivity and diet results in cardiovascular disease and metabolic complications.

High fructose corn sweeteners began widespread use in the food industry in 1967. Since that time the amount of fructose consumption has steadily risen and now accounts for about 9% of daily caloric intake in the United States. Unlike glucose, which is widely utilized by tissues throughout the body, fructose is primarily metabolized in the liver [14,15]. Recent epidemiological data suggests that high fructose corn sweeteners may be contributing to the overall epidemic of obesity and metabolic disease in the US [16].

We initiated studies to test the effects of lipoxygenase/cyclooxygenase inhibitors on the metabolic and hepatic status of HFF rats. We examined two structurally different LOX/COX inhibitors because NDGA, which has previously demonstrated effects in this model [17], also exhibits numerous other biological effects. In an initial report we showed that high levels of dietary fructose induced the JNK/AP-1 stress response pathway. This observation was confirmed by Wei and Pagliassotti, who correlated fructose-induced JNK activity with hepatic insulin resistance [18]. In our first report, we focused on the correlation between normalization of the stress response pathway and reduction of serum triglycerides. In this report, we expanded these studies to examine diet-induced effects on hepatic peroxisome proliferators-activated receptor α (PPARα) activity and the relationship between LOX/COX inhibitor treatment and of PPARα expression.

PPARα (NR1C1) is a ligand (lipid)-activated transcription factor that belongs to the superfamily of nuclear receptor [19-22]. In addition to PPARα, the NR1C subset of receptors includes two closely related members, PPARβ (or δ, NR1C2) and PPARγ (NR1C3). PPARα is highly expressed in the liver, cardiac muscle, intestine and renal cortex tissues [19,23], serves an essential function in the regulation of lipid metabolism and controls the expression of a number of genes involved in mitochondrial and peroxisomal β-oxidation [19-22]. It increases gene transcription by binding as a heterodimer with retinoid X receptor (RXR) to PPAR response elements (PPRE) on the promoter regions of the target gene [22]. Activators of PPARα, such as fibrates, lower circulating levels of lipid and are commonly used to treat hypertriglyceridemia [21,22,24]. More recent studies have shown an inverse relationship between PPAR activity and abnormalities in fatty acid metabolism leading to the development of insulin resistance and alterations in glucose metabolism [25-27]. In addition, animal studies suggest that that the activation of PPARα improved the insulin resistance that was triggered by the excessive production and accumulation of lipids [28-32].

Our studies indicate hypertriglyceridemia induced by fructose feeding greatly reduces the hepatic expression of both PPARα protein and mRNA levels. We further demonstrate that diet-induced suppression of hepaic PPARα is reversed upon treatment with lipoxygenase/cyclooxygenase inhibitory compounds. Our findings also suggest that these compounds to some extent also up-regulated the mRNA levels of ubiquitous PPARβ/δ. From these studies, we conclude that anti-inflammatory agents have the potential to prevent or treat multiple aspects of the metabolic syndrome.

## Methods

### Animals and treatments

Male Sprague-Dawley rats weighing approximately 180–200 g first maintained on a rat chow diet and then were divided into 6 groups and were switched to a high-fructose diet (TD89247; Harlan Teklad, Madison, WI) that provided 60% of total calories as a fructose (Day 1). On day 15 of treatment, the rats were fasted for 4 hours and tail vein blood was collected for baseline measurements of serum TG, glucose, insulin and free fatty acids (FFA) as previously described [33]. The groups of rats were then treated with either vehicle (0.5% carboxymethyl cellulose) or BW755c (suspended 0.5% carboxymethyl cellulose) at one of several doses ranging from 5 mg/kg to 100 mg/kg. The vehicle group and most groups receiving BW-755c or NDGA (250 mg/kg) were treated twice a day (**b.i.d.**) for 4 days, delivered by oral gavage i.e., animals were treated with vehicle or drug at day 0 at 4:00 PM, at day 1 at 9:00 AM and 4:00 PM, at day 2 at 9:00 AM and 4:00 PM, at day 3 at 9:00 AM and 4:00 PM and at day 4 at 9:00 AM. In addition, one group of animals was treated with 50 mg/kg BW-755c once a day (**q.d.**) at 9:00 AM by oral gavage. During the treatment regimen the animals were maintained on high-fructose diet. On the last day (day 4), animals were fasted for 4 h (8:00 AM-12:00 PM) and blood was collected from the tail vein 3 hours after last dose (i.e., 9:00 AM to 12:00 PM) and serum samples analysed for TG, glucose, insulin, FFA, and total cholesterol [33-35]. After the serum was collected, the animals were sacrificed and tissues removed, snap-frozen in liquid nitrogen and stored at -80°C until analysed. The local committee on animal care approved all animal protocols.

### RNA isolation and reverse transcription

The liver samples used in these studies were derived from animals treated as previously described [36].

Total RNA was extracted from the liver samples (~100–120 mg) using the Trizol reagent (Invitrogen) according to the protocols recommended by the manufacturer. Subsequently, purified RNA preparations were treated with DNAse (to eliminate the possible contamination of the genomic DNA) and further cleaned using Rneasy spin column (Qiagen, Valencia, CA). The integrity of the purified total RNA samples to be used in the Real-time PCR assays was confirmed by 1.2% formaldehyde-agarose gel electrophoresis. No degradation or 28S and 18S rRNA was observed following staining gels with ethidium bromide.

First strand cDNAs were generated from total RNA samples as follows: 2.0 μg each of total RNA sample was denatured for 5 min at 65°C in the presence random hexamer, snap cooled in ice water, then reverse transcribed in 100 μl using the TaqMan Reverse Transcription Kit (Applied Biosystems, Foster City, CA) according to the manufacturer's instructions.

### Quantitative Real-time PCR measurements

Quantitative real-time PCR (Q-PCR) amplications were performed in triplicate using the GenAmp Sequence Detection System (Applied Biosystems). The incubation mixture in a final volume of 50-μl contained suitable aliquot of template (cDNA or RNA), 300 nM each of forward and reverse primers for PPARα or 18S ribosomal RNA and 1X SYBR^® ^Green PCR Master Mix (Applied Biosystems) in 50-μl. Gene-specific primers were designed for rat PPARα gene and 18S rRNA using Primer Express software (Applied Biosystem) using sequences accessed through GenBank. The primer sequences are as shown in Table 1. Samples were amplified in an ABI Prism™ 7700 Sequence Detection System (Applied Biosystems). The change in fluorescence of SYBR^® ^Green I dye in every cycle was monitored, and threshold cycle (*C*_*T*_) above background for each reaction (i.e., the partial cycle at which statistically significant increases in either the PPARα or 18S rRNA first detected) was calculated. The calculated *C*_*T *_values for PPARα in response to various treatments were normalized to the respective *C*_*T *_values for 18S rRNA and expressed as 'Relative Values'. Initially, post-amplification melting curves and gel-electrophoresis analyses were performed to confirm that a single PCR product was produced in each reaction. The contribution of contaminating genomic DNA to the observed product in each case was determined from the *C*_*T *_given by RNA template. These values usually ranged less than 0.1% of cDNA values.

**Table 1:** Gene-specific primer used for Real Time RT-PCR assays

**GenBank **Accession **N**umber	Primer Sequence (5'→3')	Amplicon Size (bp)	Region of Gene (nt)
NM_013196	**Rat PPARα**	177	
	Forward TCACACAATGCAATCCGTTT		876–895
	Reverse GGCCTTGACCTTGTTCATGT		1052–1033

V01279	***Rat 18S rRNA***	133	
	Forward ATGGCCGTTCTTAGTTGGTG		1336–1355
	Reverse AACGCCACTTGTCCCTCTAA		1468–1449

Primer sequences were designed using Primer Express software (Applied Biosystems) using sequences accessed through GenBank. Amplicon size and region of each gene are indicated.

### Western Blot analysis of hepatic PPARα

Liver samples (~200 mg) were homogenized using a Potter-Elvehjem homogenizer in 3 volumes of detergent containing lysis buffer [20 mM HEPES, pH 7.4, 1% Triton X-100 (v/v), 150 mM NaCl, 1 mM EDTA, 1 mM EGTA, 20 mM NaF, 20 mM β-glycerophosphate, 10 mM sodium pyrophosphate, 1 mM sodium vanadate, 10 nM okadaic acid, 1 mM dithiothreitol, 10 μg/ml aprotenin, 10 μg/ml leupeptin, 10 μg/ml pepstatin A, 0.5 mM 4-(2-aminoethyl)benzylsulfonyl fluorid (AEBSF, Roche Molecular Biochemicals), 10 μM E-64 and 50 μM Bestatin] and incubated for 30 min at 4°C on an orbital shaker for complete lysis. The lysates were cleared by centrifugation at 15,000 × g for 10 min, the protein concentration of each solubilized lysate was determined and samples stored frozen until analyzed.

Samples containing an equal amount of protein (50 – 60 μg) were fractionated by SDS-polyacrylamide gel electrophoresis (10% polyacrylamide gel with 4% stacking gel) and transferred to polyvinyllidene difluoride membrane (Immobilon™, Millipore Corp., Bedford, MA). After transfer the membranes were stained with Ponceau S dye (Sigma Chemical Co. St. Louis, MO) to verify loading equivalency and transfer efficiency and then the membrane was washed in TBS containing 0.1% Tween-20 (TTBS) and incubated in blocking buffer (TTBS containing 5% non-fat dry milk) for 90 min at room temperature followed by overnight incubation at 4°C with rabbit anti-PPARα IgG (Santa Cruz Biotechnology, Santa Cruz, CA) diluted in blocking buffer. Subsequently, the membrane was washed in TTBS and incubated for 2 hr with horseradish peroxidase conjugated goat anti-rabbit IgG (Sigma Chemical Co. St. Louis, MO) in blocking buffer. Bands were visualized by enhanced chemiluminescence detection as described by the manufacturer (ECL System, Amersham Pharmacia Biotech). Blots were exposed to film for various times (3–10 min), and exposures were subjected to densitometric scanning using Fluor-S™ MultiImager with a built-in computer software (Bio-Rad). Equal loading of proteins was confirmed by staining the membranes with the Ponsceau S.

### Statistical Analysis

Dose response analysis was conducted using paired t-tests. PPARα RT-PCR and Western Blots were analyzed using t-test comparing the drug treatment group to the vehicle control. All statistical analyses were performed using GraphPad Prism version 3.00 for Windows, GraphPad Software, San Diego California USA, ****.

## Results

### Dose-responsive reduction of diet-induced hypertriglyceridemia

**Figure 1:**
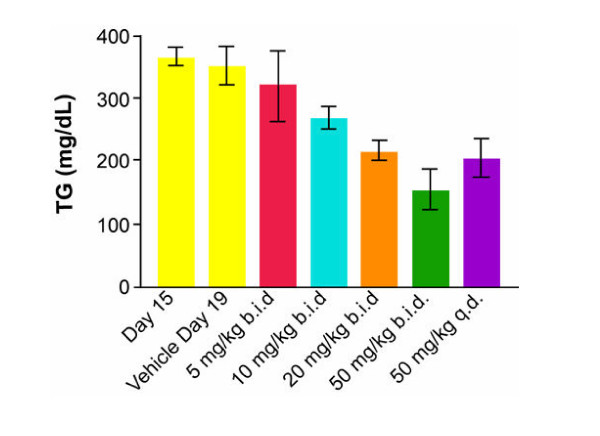
**Dose-responsive reduction of serum triglycerides upon treatment with BW-755c (mean +/- SD)**. The statistical significance of each of the dose groups is shown in Table 2.

Table 2 summarizes the effect of 4 consecutive day's treatment using BW-755c on HFF animals. During the treatment phase no obvious toxic effects were noted in the animals. All animals at all dose groups gained weight during the testing period. In these animals there was no discernable hyperinsulinemia or elevated FFA. The only metabolic effect in this group of animals was hypertriglyceridemia. BW-755c lowered serum triglycerides in a dose-related fashion (Figure 1). In addition, there was no difference between dosing the animals once daily versus twice daily as can be appreciated by comparing the 20 mg/kg bid (40 mg/kg daily) results versus the 50 mg/kg qd groups. There were no changes in any of the other measured metabolic parameters during the test period.

**Table 2:** Dose-responsive effect of BW-755c on hypertriglyceridenia in HFF rats

	HFF Day 15 n = 24	Vehicle n = 4	5 mg/kg BW (b.i.d.) n = 4	10 mg/kg BW (b.i.d.) n = 4	20 mg/kg BW (b.i.d.) n = 4	50 mg/kg BW (b.i.d.) n = 4	50 mg/kg BW (q.d.) n = 4
Weight (g)	301 ± 2.3	312 ± 8^a^	312 ± 7^a^	312 ± 5^d^	310 ± 5^a^	316 ± 6^b^	317 ± 6^c^
Glucose (mg/dl)	116.0 ± 1.6	113.0 ± 3.4	113.8 ± 6.4	115.3 ± 5.1	112.8 ± 2.9	115.5 ± 8.7	119.3 ± 7.0
Insulin (ng/ml)	1.84 ± 0.06	1.78 ± 0.13	1.83 ± 0.13	1.86 ± 0.15	1.77 ± 0.09	1.82 ± 0.05	1.81 ± 0.13
FFA (μEq/l)	532 ± 17	553 ± 54	552 ± 63	543 ± 92	528 ± 32	562 ± 39	540 ± 52
Cholesterol (mg/dl)	109.6 ± 1.5	106.8 ± 5.8	109.5 ± 6.4	105.0 ± 3.5	107.8 ± 2.8	109.0 ± 4.1	113.3 ± 3.2
TG (mg/dl)	367 ± 16	351 ± 31	319 ± 59	268 ± 18	216 ± 17^a^	154 ± 33^a^	215 ± 39^a^

Results are Mean ± SE

^a^*p *< 0.001; ^b^*p *< 0.002; ^c^*p *< 0.004; ^d^*p *< 0.04

### BW-755c treatment restores PPARα protein levels in HFF animals

**Figure 2:**
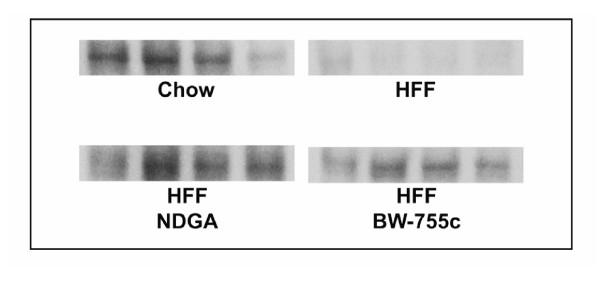
**Representative Western blots for PPARα expression in hepatic tissue from chow-fed (control) rats and high fructose-fed rats that were subsequently treated with vehicle alone, NDGA or BW755c**. Each lane represents hepatic tissue from each of four different animals in each group.

Figure 2 shows that the amount of hepatic PPARα protein is reduced upon initiation of the HFF diet. Each lane represents a liver sample from a different animal in the dosing group. This was done to control for variation due to unrestricted feeding of the animals and it's potential impact on the expression of PPARα and to ensure that the observed responses were generalized to the group and not an animal-specific observation. Liver samples from chow-fed animals were included as a control group because they represent normal expression levels of PPARalpha. Liver samples from the group of vehicle treated HFF animal represent the expected degree of diet-induced PPARalpha suppression. Liver samples from HFF animals that were treated with either NDGA or BW-755c demonstrate normal or elevated levels of PPARα protein (Table 3). This data shows that these compounds are able to overcome the diet induced suppression either directly or by a compensatory mechanism.

**Table 3:** Quantitative Western blot/densitometric analysis of hepatic PPARα protein levels

Diet	Treatment	PPARα level (Mean ± SE)
Normal Chow	Vehicle	0.149 ± 0.025
HFF	Vehicle	0.038 ± 0.005^a^
HFF	NDGA-250 mg/kg (b.i.d.)	0.290 ± 0.050^b^
HFF	BW-755c-100 mg/kg (b.i.d.)	0.190 ± 0.030^c^

Results are Mean ± SE of four different samples

^a^*p *< 0.004 Chow *vs *HFF

^b^*p *< 0.003 HFF *vs *HFF/NDGA treatment

^c^*p *< 0.003 HFF *vs *HFF/BW-755c treatment

### BW-755c treatment restores PPARα RNA expression in HFF animals

In order to determine the mechanism by which the HFF diet suppressed PPARa we measured the relative amount of PPARα RNA in the livers of the animal in the various treatment groups. As seen in Table 4, PPARα RNA is suppressed in HFF animals, indicating that the method of control is related to transcription rather than protein expression. The LOX/COX drug treatment increased PPARα RNA levels. In the case of BW-755c the relative amounts of RNA were comparable to that of the vehicle control. NDGA treatment resulted in higher levels of PPARα RNA. This may be a secondary effect of this compound because hepatic RNA levels of PPARβ/δ were also elevated in response to this drug (data not shown).

**Table 4:** Real-Time RT-PCR analysis of PPARα gene expression in liver tissue from chow-fed (control), and high fructose-fed animals treated with vehicle, NDGA or BW-755c

Diet	Treatment	PPARα normalized to 18S rRNA (Relative value ± SE)
Normal Chow	Vehicle	2.375 ± 0.576
HFF	Vehicle	0.425 ± 0.085^a^
HFF	NDGA-250 mg/kg (b.i.d.)	3.600 ± 0.339^b^
HFF	BW-755c-100 mg/kg (b.i.d.)	2.150 ± 0.417^c^

Results are Mean ± SE of four different samples

^a^*p *< 0.01 Chow *vs *HFF

^b^*p *< 0.001 HFF *vs *HFF/NDGA treatment

^c^*p *< 0.001 HFF *vs *HFF/BW-755c treatment

## Discussion

There is building evidence that improper consumption of metabolic components, including fatty acids and carbohydrates, can lead to systemic inflammation. This systemic inflammation is at least contributory, if not causative, to metabolic disease progression.

The early stage of the HFF model is useful to examine dislipidemia because the animals lack many confounding factors such as insulin resistance or the impact of elevated FFA or obesity. In the earliest stage of metabolic dysfunction, these animals receive excess dietary fructose and exhibit hypertriglyceridemia. Hepatic fructose metabolism leads to precursors of triglyceride synthesis and thus it is not altogether surprising that dietary fructose leads to generation of triglycerides. Clearly, however the compensatory metabolic regulatory mechanisms are disrupted in these animals because they fail to control triglycerides within normal ranges. In our prior work, we showed a correlation between activation of the JNK stress pathway leading to activation of the transcription factor AP-1 and abnormal triglyceride levels. We also showed that LOX/COX inhibitors inhibited the JNK/AP-1 activation and correspondingly reduced serum triglyceride levels even in the face of excess dietary fructose.

In this paper we extend this correlation to the expression of PPARα, in which excess dietary fructose suppresses PPARα but such suppression can be overcome by administration of these LOX/COX inhibitory compounds. The PPARα is lipid activated transcription factor that plays a pivotal role in the transcription regulation of genes involved in lipid catabolism and lipoprotein metabolism. In hepatocytes and other tissues (e.g., heart) natural long chain fatty acids (ligand) activated PPARα binds to peroxisome proliferators response element (PPRE) of DNA and increases the transcription of genes encoding enzymes involved in fatty acid oxidation (e.g., acyl-CoA oxidase and carnitine palmitoyltransferase) and lipoprotein (HDL and VLDL/TG) metabolism (e.g., apo-AI, AII, AV, CIII, and PTP and LPL) [19,37,38]. The out come is an increase in hepatic fatty acid oxidation and ketogenesis, decreased tissue levels of lipids and protection against lipotoxicity. Our present data suggest that expression of PPARα activity is primarily regulated at the level of transcription as determined by comparing RNA levels of hepatic PPARα in chow-fed versus HFF animals. The fact that the LOX/COX inhibitors resulted in restored levels of hepatic PPARα RNA suggests that these compounds are influencing signalling pathways that regulate PPARα transcription. Beier et al have shown that TNFα downregulates expression of hepatic PPARα RNA [39]. We and others have demonstrated that the HFF diet induces a TNFα-like stress response through the JNK pathway [18,36]. Therefore a likely mechanism by which hypertriglyceridemia is elicited in the HFF model is as follows:

1. Consumption of high levels of dietary fructose leads to activation of the JNK/AP-1 stress response pathway.

2. The activation of the stress response suppresses PPARα expression in the liver;

3. Suppression of PPARα disrupts normal lipid homeostasis and metabolism;

4. The metabolism of fructose leads to an abundance of TG precursors that provide a source for TG synthesis; and

5. The fructose-mediated stress response may increase the expression of sterol regulatory element-binding protein-1c (SREBP-1c) [40,41], which activates the genes involved in this seems to be unlikely possibility given the fact the cytokine TNFα is known to negatively regulate SREBP-1c expression [42,43] and that expression of lipogenic enzymes is also achieved through an SREBP-1c independent mechanism [44].

With this as a working model, the mechanism by which the LOX/COX inhibitors could act would be by their anti-inflammatory actions in the TNFα pathway. By preventing activation of the stress response pathways, PPARα levels are not suppressed and therefore lipid homeostasis would not be disrupted. Therefore the excess fructose can be normally metabolized in other ways, such as being shunted into gluconeogenesis pathways.

## Conclusion

The current studies indicate that hypertriglyceridemia induced by high fructose feeding leads to major reduction in the steady-state levels of both PPARα protein and mRNA. Treatment with lipoxygenase/cyclooxygenase inhibitor compounds reversed the fructose-induced suppression of hepatic PPARa expression. These compounds also up-regulated mRNA levels of ubiquitous PPAR isoform, PPARβ/δ. These anti-inflammatory agents have the therapeutic potential in the prevention and/or management of various aspects of the metabolic syndrome.

## List of Abbreviations

AP-1 – Activator Protein-1

BID – Twice daily

BW-755c – 4,5-Dihydro-1-(3-(trifluoromethyl)phenyl)-1H-pyrazol-3-amine

COX – cyclooxygenase

HFF – high fructose-fed diet

JNK – Janus Kinase

LO – lipoxygenase

NDGA – nordihydroguaiaretic acid

PPAR – peroxidase proliferators-activated receptor

TG – triglyceride

TNF – Tumor Necrosis Factor

SREBP-1c – sterol regulatory element-binding protein-1c

## Competing interests

The author(s) declare that they have no competing interests.

## Authors' contributions

Both authors declare that they have made contributions to experimental design and analysis/interpretation of the data. Both authors have been involved in drafting the manuscript and have given their approval for the publication of this manuscript.
